# Depression among psychiatrists and psychiatry trainees and its associated factors regarding work, social support, and loneliness

**DOI:** 10.1186/s12888-024-05569-7

**Published:** 2024-02-05

**Authors:** Jarurin Pitanupong, Kanthee Anantapong, Warut Aunjitsakul

**Affiliations:** Department of Psychiatry, Faculty of Medicine, Prince of Songkla University, Hat Yai, Songkhla 90110 Thailand

**Keywords:** Associated factors, Depression, Prevalence, Psychiatrist, Psychiatry trainee

## Abstract

**Background:**

This study aimed to survey the prevalence of depression and its associated factors among psychiatrists and psychiatry trainees (physicians in psychiatric residency training).

**Methods:**

This cross-sectional study surveyed Thai psychiatrists and psychiatry trainees from January to February 2023 using an online questionnaire. The questionnaires consisted of (1) the demographic and work-related information; (2) perceptions towards social support and work; (3) the Patient Health Questionnaire-9 (PHQ-9) Thai version; and (4) the 6-item Revised UCLA Loneliness Scale Thai version. All data were analyzed using descriptive statistics, and the associated factors concerning depression were analyzed via multiple linear regression analyses.

**Results:**

Of the 225 total participants, 52(23.1%) and 173 (76.9%) were psychiatry trainees and psychiatrists, respectively. Most of them were female (64.9%) with overall median age (interquatile) was 34 (30, 42) years. Regarding the PHQ-9 findings, the prevalence of depression among all participants was 12.4% (psychiatrists 13.9% and psychiatry trainees 7.7%). From regression analyses, depression was associated with loneliness and perceived levels of work satisfaction and work stress in psychiatrists, while in psychiatry trainees, depression was associated with loneliness and perceived level of ability to control work schedule.

**Conclusions:**

One-tenth of psychiatrists and psychiatry trainees had depression. Although the prevalence of depression in this study was not extremely high, key contributing factors related to depression, such as loneliness, work satisfaction, work stress, and ability to control the work schedule should be required to action to reduce the depression rate among mental health personnel.

**Supplementary Information:**

The online version contains supplementary material available at 10.1186/s12888-024-05569-7.

## Background

Major depressive disorder (MDD) is a common illness [1] that has a negative impact on the afflicted individual as a consequence of altered functioning [2]. Previous studies reported that prevalences of MDD, depending on the instrument used, were 10.8–23.2% among physicians [3–5], and 20.9–43.2% among resident physicians [6].

A prior cross-sectional, multi-country online survey among psychiatry trainees in European countries reported that the prevalence of MDD was approximately 20.8%, using the Patient Health Questionnaire-9 (PHQ-9) [7]. Another study using the PHQ-9 reported that 16.1% of North American psychiatrists had MDD [8]. In Thailand, the number of psychiatrists remains short and relatively small compared to those found in more developed countries [9]. In 2016, numbers of psychiatrists per 100,00 people in countries such as the USA, Japan, or New Zealand were 10.54, 11.87 or 28.54, respectively, on the other hand in Thailand was 0.72 [10]. Thai psychiatrists reported low quality of life (QoL) [11] and suffered from high levels of burnout, increasing dramatically from 17.1 to 49.3% (national surveys of Thai psychiatrists in 2011 [12] and 2018) [13].

Among the factors associated with MDD among psychiatrists, female gender, being in residency training or one’s early-career stage, practicing in a nonacademic setting, and burnout were significantly related to higher PHQ-9 scores or the presence of MDD [8]. Additionally, working more than 50 h per week, a higher caseload or caring for more patients per day [13, 14], feeling unsatisfied with one’s work and environment, and receiving insufficient support from family, workplace, and colleagues were associated with higher levels of burnout [13] and lower QoL [11]. Several studies investigate MDD mostly in psychiatrists, but less in psychiatry trainees; and mostly about burnout and quality of life, but less in work-related data, which may link to emotional distress. The factors related to work included, for example: perception of social support towards family, chief of department, and psychiatrist/psychiatry trainee friends; or perceptions towards work including ability to control one’s work schedule, work stress, satisfaction with one’s income and work [13, 14]. Furthermore, loneliness – an indicator of social well-being – can lead to various psychiatric disorders and various physical disorders [15], which higher level of loneliness could lead to poorer MDD, not only social support but development of psychological interventions required for MDD [16].

During the Coronavirus disease (COVID-19) pandemic, psychiatry colleagues around the world have faced mental health problems associated with the pandemic of patients and their families (such as anxiety, and panic) [17]. This outbreak still substantial challenges to psychiatry as it requires providing diagnostic and therapeutic services where and when they are needed [17], but there are not enough psychiatrists to meet this demand [18]. As a consequence of the pandemic, psychiatry, and mental health professionals have been negatively affected, their distresses linked to clinical work and pressures from personal lives. However, psychological supports were offered and provided just little to them [18].

Thus, this study was conducted in both psychiatrists and psychiatry trainees in Thailand to examine the prevalence of MDD. Associating factors of work related to MDD were also investigated because the factors have not yet been fully investigated among psychiatrists and psychiatry trainees. The findings from this study would be valuable to inform the relevant stakeholders on how to promote mental health of Thai psychiatrists and psychiatry trainees.

## Methods

Following the ethical principles of the Declaration of Helsinki, this survey was approved by the Human Research Ethics Committee of the Faculty of Medicine, Prince of Songkla University (REC.65-488-3-1). This study was a cross-sectional, online-based survey from January to February 2023.

### Participants

Sample size calculation, from the literature review, there was limited evidence of the prevalence of MDD among psychiatrists. The prior study reported the prevalence of MDD among psychiatrists was 16.1% [8]. There were approximately 123 Thai psychiatry trainees and 499 psychiatrists who were members of the Royal College of Psychiatrists of Thailand. The function ‘n.for.survey’ from the Epicalc package in the R program was used for the calculation of the sample size for a finite population. Given delta was 0.04, the alpha was 0.05, and the population size was 622, therefore the sample size required was at least 197. We recruited data from all Thai psychiatry trainees and psychiatrists as available for the convenient management by announcement via the official social media platform of the Thai Royal College of Psychiatrists e.g., Facebook, Line application (a freeware application for instant communications on electronic devices operated by LY Corporation). The participants, who were aged between 20 and 70 years and able to use the Thai language, were included.

### Data collection

Participants were recruited with a non-probability sample from the official social media platform of the Thai Royal College of Psychiatrists. We have advertised the survey via online platforms using a poster with QR code and/or link. The poster provides information related to brief introduction and objectives of the study. The participants who were interested in collaborating with the survey entered through the link or QR code from advertisements. On the first page, all participants were provided with the rationale and an overview of the survey to consider whether or not to take part in the study. If they agreed to participate in the survey, they then clicked continue to fill out the questionnaire. Adhering to the policy of strict confidentiality, the signatures of the participants were not required, and all of them were advised that they retained the right to withdraw from the survey at any time without giving any reason.

### Measurements

The demographic data, e.g., age, gender, marital status, number of children in the family, and history of physical and psychiatric illness were collected. Work-related data were also collected, including career status (psychiatry trainees vs. psychiatrists), type of psychiatrist (general psychiatrist vs. child and adolescent psychiatrist), type of workplace (academic medical school vs. psychiatric hospital vs. general/community/private hospital or clinic), type of patient’s care (outpatient vs. both outpatient and inpatient care) as well as: experience years as a psychiatrist or psychiatry trainee; number of patients seen per day; clinical hour per week; night shifts per month; paid days off per month; and death patients who committed suicide. In addition, we collected perceived social support, whether poor or good, provided by family, the head of the department, and peers. To explore the perception towards work, we used a visual analog scale (VAS), ranging from 1 to 10 (extremely low – extremely high) to document perception levels of ability to control one’s work schedule; work stress; and satisfaction with one’s income and work. Additionally, two instruments Thai version were used in this study, one for the measurement of depression and the other for loneliness, as follows.

### Depression

The Patient Health Questionnaire-9 (PHQ-9) Thai version is a self-rating questionnaire to evaluate depression, which consists of 9 items. It employs a 4-point rating scale for each question: 0 (never), 1 (rarely), 2 (sometimes), and 3 (always). The total score can range from 0 to 27; 0–4 (no or minimal depression), 5–9 (mild depression), 10–14 (moderate depression), 15–19 (moderately severe depression), and 20–27 (severe depression). The PHQ-9 Thai version has been proven to have acceptable psychometric properties for the screening of MDD with a recommended cut-off score of nine or greater, with a sensitivity of 0.53 and a specificity of 0.98 and Cronbach’s alpha coefficient of 0.79 [19]. In this study, the questionnaire has demonstrated the Cronbach’s alpha coefficient value of 0.81.

### Loneliness

The 6-item Revised UCLA Loneliness Scale (RULS-6) Thai version is a self-rating questionnaire used to evaluate loneliness. It employs a 4-point rating scale for each question: 1 (never), 2 (rarely), 3 (sometimes), and 4 (always). The total score ranges from 6 to 24. The higher the score, the greater the degree of loneliness is indicated. The summed scores were grouped into three categories: ‘low’ for a score below the 25th percentile, ‘average’ for scores ranging from the 25th to the 75th percentiles, and ‘high’ for a score above the 75th percentile. The questionnaire has demonstrated good internal consistency as indicated by a Cronbach’s alpha coefficient of 0.83 [20]. Regarding the data from this study, the questionnaire has demonstrated the Cronbach’s alpha coefficient value of 0.87.

### Statistical analyses

All data (e.g., demographic and work-related data and variables of interest) were analyzed using the descriptive statistic method comprising proportions, means, standard deviation (SD), median, and interquartile range (IQR) and using Pearson’s or Spearman’s correlation analyses for inter-correlation of variables, according to normal or non-normal distribution. Based on Shapiro-Wilk test for normality, if the results indicated that *p* < 0.05, suggesting that the assumption of normal distribution was violated, therefore, the Spearman’s correlation analyses will be used to calculate correlation coefficient. For data management, any missing value of variable higher than 10% would be grouped into another category for data analyses, no data are removed from the statistical analyses. The association between depression score and demographic data, work-related factors, and variables of interest were analyzed using multiple linear regressions with backward elimination. Regarding an input for the multivariate analysis, the variables were selected from univariate analyses, which *p*-value should less than 0.2. To address potential confounding effects, we carefully identified and considered potential confounders, based on both existing literature and the variables of interest. These variables were then added as covariates in the multiple linear regression model. Variance Inflation Factors (VIFs) were calculated for each variable in the model to assess multicollinearity among candidate variables, if the VIF value was less than 5, indicating low correlation among each other variables. The likelihood ratio (LR) test was conducted to assess the model fit, which significant *p*-value of LR test was 0.05. All analyses were performed using R version 4.1.2 (R Foundation for Statistical Computing). All confidence intervals (CIs) were calculated at the 2-sided 95% level.

## Results

### Demographic characteristics

225 participants agreed to collaborate and complete the questionnaires, comprising 173 (76.9%) psychiatrists and 52 (23.1%) psychiatry trainees. The response rate was 36.2%. The median (IQR) age among the psychiatrists and psychiatry trainees was 37 (33, 44) and 28 (27, 29) years old, respectively. The majority of the respondents were female (149, 64.9%), single/divorced (146, 64.9%), and had a previous history of physical (91, 40.4%) and psychiatric illnesses (19, 8.4%). The most common physical illnesses were allergies (10.2%) and dyslipidemia (7.6%), while psychiatric illnesses were MDD (6.2%), generalized anxiety disorder (1.4%), post-traumatic stress disorder (0.4%) and attention deficit hyperactivity disorder (0.4%). Regarding work-related characteristics, most participants were general psychiatrists (178, 79.1%), worked at a medical school (105, 46.7%), and worked from 40 to 50 h per week (108, 48.0%), other details see Table 1.

**Table 1:** Demographic, medical and work-related characteristics (*N* = 225)

Variables	Total (*N* = 225); N (%)	Psychiatrists (*n* = 173); n (%)	Psychiatry trainees (*n* = 52); n (%)
**Gender**			
Male	79 (35.1)	64 (37.0)	15 (28.8)
Female	146 (64.9)	109 (63.0)	37 (71.2)
**Age (years)**			
Median (IQR)	34 (30, 42)	37 (33, 44)	28 (27, 29)
**Marital status**			
Single/Divorced	146 (64.9)	99 (57.2)	47 (90.4)
Married	79 (35.1)	74 (42.8)	5 (9.6)
**Number of children**			
None	176 (78.2)	125 (72.3)	51 (98.1)
One	29 (12.9)	28 (16.2)	1 (1.9)
More than one	20 (8.9)	20 (11.6)	0 (0.0)
**Physical illness**			
No	134 (59.6)	100 (57.8)	34 (65.4)
Yes	91 (40.4)	73 (42.2)	18 (34.6)
**Psychiatric illness**			
No	206 (91.6)	159 (91.9)	47 (90.4)
Yes	19 (8.4)	14 (8.1)	5 (9.6)
**Experience as a psychiatrist (years)**			
Median (IQR)	6 (3, 14)	9 (5, 15)	2 (1, 3)
**Type of Workplace**			
Medical school	105 (46.7)	60 (34.7)	45 (86.5)
Psychiatric hospital	24 (10.7)	17 (9.8)	7 (13.5)
Other hospital (general/community/private hospital/clinic)	96 (42.7)	96 (55.5)	0
**Types of a psychiatrist**			
General psychiatrist	178 (79.1)	138 (79.8)	40 (76.9)
Child and adolescent psychiatrist	46 (20.4)	35 (20.2)	11 (21.2)
No answer	1 (0.4)	0	1 (1.9)
**Types of patient’s care**			
Only outpatient	22 (9.8)	22 (12.7)	0 (0.0)
Outpatient and inpatient	203 (90.2)	151 (87.3)	52 (100)
**Number of patients per day**			
Median (IQR)	20 (10, 30)	25 (14, 35)	10 (6, 15)
**Working hours per week**			
<40	57 (25.3)	51 (29.5)	6 (11.5)
40–50	108 (48.0)	80 (46.5)	28 (53.8)
>50	59 (26.2)	41 (23.7)	18 (34.6)
No answer	1 (0.4)	1 (0.6)	0
**Number of night shifts per month**			
Median (IQR)	6 (4, 10)	6 (2.8,10)	5.5 (4,9.2)
**Number of days off per month**			
0–2	21 (9.3)	13 (7.5)	8 (15.4)
3–5	48 (21.3)	39 (22.5)	9 (17.3)
6–8	101 (44.9)	68 (39.3)	33 (63.5)
8–10	46 (20.4)	44 (25.4)	2 (3.8)
>10	9 (4.0)	9 (5.2)	0 (0.0)

IQR interquartile range

### Depression

**Fig. 1:**
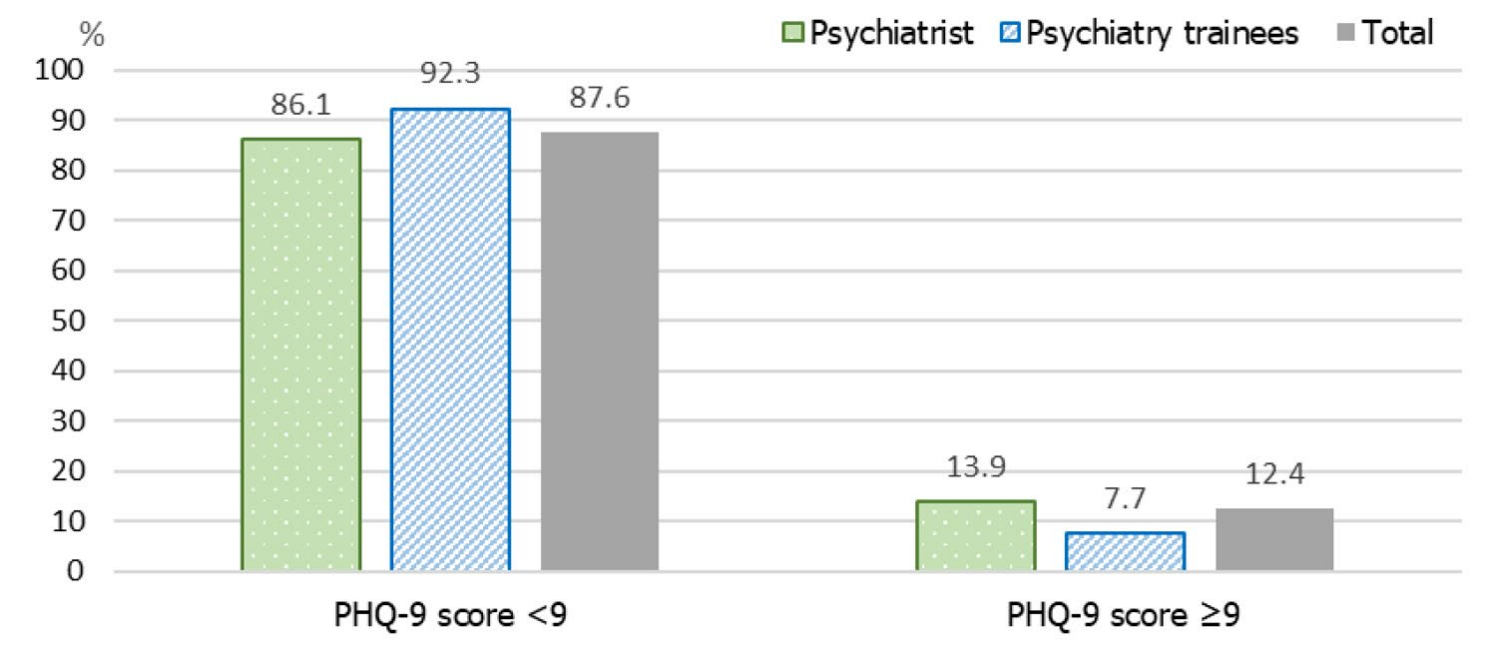
The presence of depression among psychiatry trainees and psychiatrists (*N* = 225)

Of all participants, the median (IQR) for the PHQ-9 score was 3 (2, 6). Most participants reported no/minimal (143, 63.6%), mild (63, 28.0%), and moderate to severe depression (19, 8.4%) (Supplementary Table 1). 28 (12.4%) participants were considered as MDD due to their PHQ-9 score ≥ 9, including 24 (13.9%) psychiatrists and 4 (7.7%,) psychiatry trainees (Fig. 1). Additionally, among those with MDD, there were 3 psychiatrists and 1 psychiatry trainee who had a previous history of MDD. Categorized data by the PHQ-9 with other variables are shown in Supplementary Table 2.

**Fig. 2:**
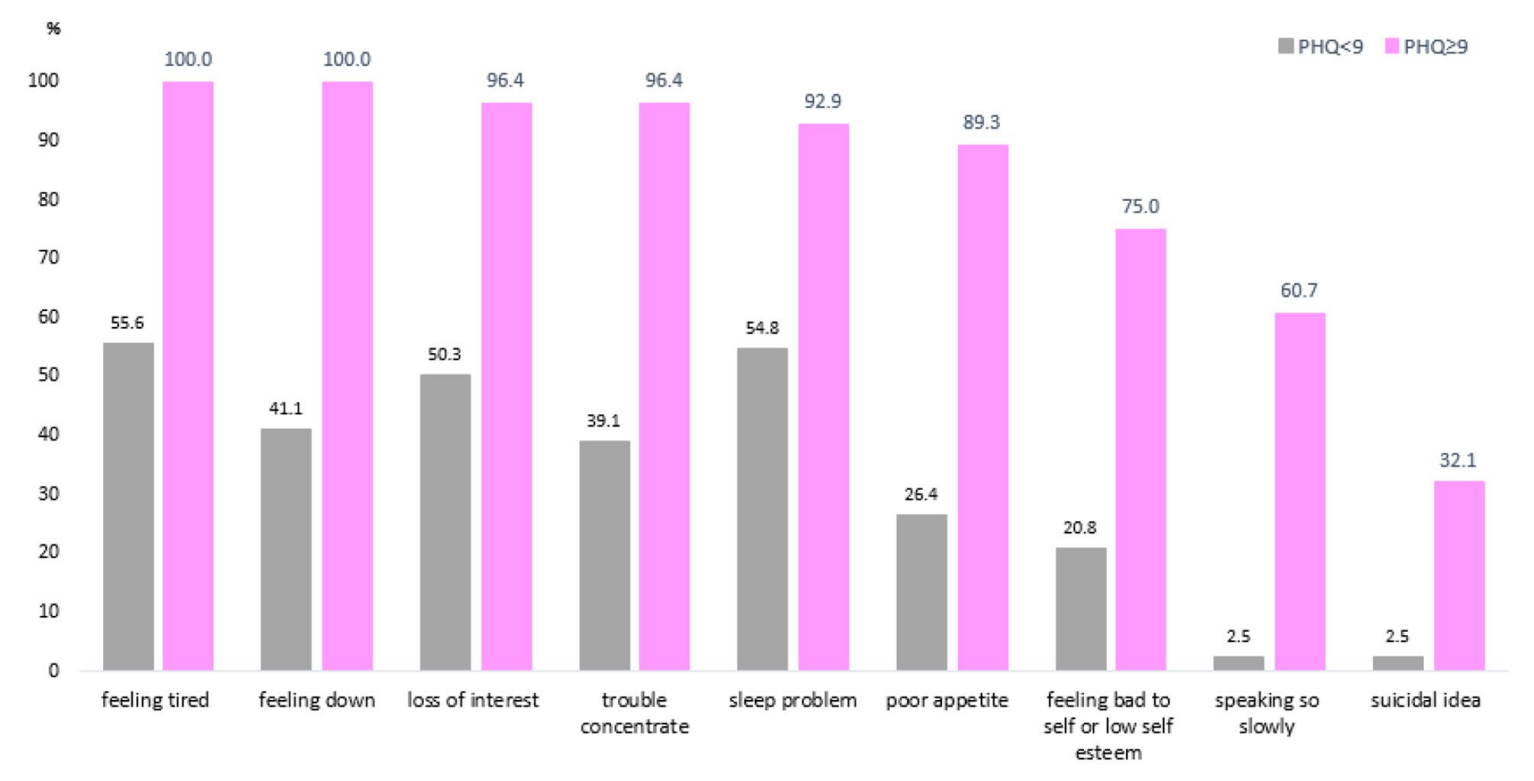
The frequency of symptoms of depression according to the depression (*N* = 28) and non-depression status (*N* = 197)

Regarding the symptoms of MDD according to the PHQ-9 findings, feeling down, feeling tired, lack of pleasure, having trouble concentrating, and the presence of sleep problems were the most frequently reported symptoms, see Fig. 2. Regarding suicidal ideation, 14 participants were reported, and 9 (32.1%) of them were considered as MDD in this study.

### Loneliness

Using the RULS-6, only 35 (15.6%) participants were found to experience a high level of loneliness, while the rest reported an average level (190, 84.4%), with the proportions of those with a high level among psychiatrists and psychiatry trainees were 17.3% and 9.6%, respectively (Supplementary Table 1). Among 14 participants with suicidal ideation, 5 of them experienced a high level of loneliness, the others reported an average level. There was no statistically significant association between the presence of suicidal ideation and loneliness.

### Perception towards social support and work

Asking whether ‘poor’ or good’ about one’s perceived social support, most participants reported that they received good support from family, peers, and the head of the department were 199 (88.4%), 178 (79.1%) and 149 (66.2%), respectively. No significant difference in the social support between psychiatrists and psychiatry trainees was found.

Meanwhile, self-rating perceptions towards works from 1 (extremely low) to 10 (extremely high), mean (SD) of participants’ perceptions were 5.9 (2.2), 6.0 (2.5), 6.4 (1.9) and 7.0 (1.8) of work stress, income’s satisfaction, ability to control work schedule and work satisfaction, respectively. Their medians (IQR) and their comparisons were shown in Supplementary Table 1. Perception of income satisfaction, ability to control work schedule, and work stress were statistically significant different between psychiatrists and psychiatry trainees (*p* < 0.001, 0.023, and 0.045, respectively).

### Association of levels of depression with demographic, work-related characteristics, level of loneliness and perceptions towards social supports and works

Any variable having a statistically significant univariate test result, which was based on a *p*-value of < 0.2, was selected as a candidate for inclusion in the multivariate analysis. The crude analysis revealed a statistically significant association between the levels of depression with age, number of children, psychiatric illness, number of experience years as a psychiatrist/psychiatry trainee, number of paid days off per month, and number of death patients who committed suicide (Supplementary Table 3). Also, the level of depression significantly positively correlated with loneliness among all participants (*r* = 0.43, *p* < 0.001) with psychiatrists and psychiatry trainees were 0.47 (*p* < 0.001) and 0.30 (*p* = 0.029), respectively. Among perceptions towards work, all participants’ levels of depression only positively correlated with work stress (*r* = 0.46, *p* < 0.001), the others showed negative correlations: work satisfaction (*r*=-0.42, *p* < 0.001), ability to control work schedule (*r*=-0.34, *p* < 0.001) and income’s satisfaction (*r*=-0.20, *p* = 0.002). Furthermore, the correlation coefficients of psychiatry trainees were generally weaker than those of psychiatrists. (Table 2)

**Table 2:** Corrrelation between level of depression (using PHQ-9) and loneliness and perception towards works

Variables	Depression score					
	Total (*N* = 225)		Psychiatrists (*n* = 173)		Psychiatry trainees (*n* = 52)	
	ρ	*p*-value	ρ	*p*-value	ρ	*p*-value
**Loneliness score**	0.43	< 0.001	0.47	< 0.001	0.30	0.029
**Perception levels of**:						
Ability to control work schedule	-0.34	< 0.001	0.34	< 0.001	-0.38	0.005
Work stress	0.46	< 0.001	0.52	< 0.001	0.24	0.100
Income satisfaction	-0.20	0.002	-0.25	0.001	-0.13	0.368
Work satisfaction	-0.42	< 0.001	-0.45	< 0.001	-0.33	0.016

ρ = Spearman correlation coefficient; IQR: Interquartile range; PHQ-9: Patient Health Questionnaire-9

Note: Perception towards works using self-rating from 1 to 10 (extremely low – extremely high) of each item, representing continuous data for correlation analyses

The multicollinearity among the independent variables was checked, the calculated VIF values were from 1.03 to 1.87, indicating low multicollinearity. The results of the multiple linear regression showed the level of depression was found to be significantly associated with the presence of previous psychiatric illness (Adjusted coefficients 1.44 (0.04, 2.84; *p* = 0.043)), loneliness score (0.3 (0.17, 043; *p* < 0.001)), perceived levels work satisfaction (-0.43 (-0.68, -0.17; *p* = 0.001)) and work stress (0.43 (0.23, 0.63; *p* < 0.001)) in all participants. When analyzed separately, in psychiatrists’ group loneliness score (0.29; 0.14, 0.44; *p* < 0.001), perceived levels of work satisfaction (-0.46; -0.78, -0.15; *p* = 0.004) and work stress (0.47; 0.23, 0.71; *p* < 0.001) remained significantly associated with the level of depression. Meanwhile, in the psychiatry trainees’ group loneliness score (0.28; 0.03, 0.53; *p* = 0.029) and perceived level of ability to control work schedule (-0.49; -0.91, -0.06; *p* = 0.027) were significantly associated with level of depression after adjusting for days off per month. (Table 3)

**Table 3:** Linear regression analyses of level of depression (using PHQ-9) on variables among all participants, psychiatrists and psychiatry trainees

Variables	PHQ-9 score								
	**Total** (*N* = 220)			**Psychiatrists** (*n* = 171)			**Psychiatry trainees** (*n* = 50)		
	Crude coefficient (95% CI)	Adjusted coefficient (95% CI)	*p*-value (F-test)	Crude coefficient (95% CI)	Adjusted coefficient (95% CI)	*p*-value (F-test)	Crude coefficient (95% CI)	Adjusted coefficient (95% CI)	*p*-value (F-test)
Presence of psychiatric illness	1.64 (-0.06, 3.33)	1.44 (0.04, 2.84)	0.043						
Loneliness score	0.47 (0.34, 0.6)	0.3 (0.17, 0.43)	< 0.001	0.50 (0.35, 0.65)	0.29 (0.14,0.44)	< 0.001	0.38 (0.12, 0.64)	0.28 (0.03, 0.53)	0.029
Perception level of ability to control work schedule							-0.54 (-0.99, -0.1)	-0.49 (-0.91, -0.06)	0.027
Perception level of work satisfaction	-0.87 (-1.11, -0.63)	-0.43 (-0.68,-0.17)	0.001	-0.98 (-1.27, -0.69)	-0.46 (-0.78, -0.15)	0.004			
Perception level of work stress	0.71 (0.52, 0.91)	0.43 (0.23,0.63)	< 0.001	0.80 (0.58, 1.02)	0.47 (0.23, 0.71)	< 0.001			

CI: Confidence interval; PHQ-9: Patient Health Questionnaire-9

Note: Adjusted R-square of models: all participants, psychiatrists and psychiatry trainees were of 0.33, 0.34 and 0.32, respectively

## Discussion

This is the first national survey from Thailand aimed to explore the prevalence and associated factors of MDD among Thai psychiatrists and psychiatry trainees. Regarding the objectives, this study found that 13.9% of psychiatrists and 7.7% of psychiatry trainees were considered MDD. Higher loneliness score was associated with higher depression scores in both psychiatrists and psychiatry trainees. In psychiatrists, a high depression score was associated with a low perceived level of work satisfaction and a high perceived level of work stress, whereas in psychiatry trainees’ a high depression score was associated with a low perceived level of ability to control work schedule.

As for the prevalence of MDD, the finding from this study of 13.9% of psychiatrists was found to be relatively comparable with the prior study in North America which 16.1% were identified as MDD [8]. This finding was different from the prior report, a potential explanation for these discrepancies may be due to different study instruments, and characteristics of the population; such as age and socioeconomic differences, and ethnicity.

Meanwhile, among psychiatry trainees in this study, the prevalence of MDD (7.7%) was different from prior studies with an average rate of 20.8%, nevertheless, there was a wide range of MDD prevalences from 7.1 to 50.0% depending on the country site [7]. A recent study among Thai residents found that 23.4% of them had MDD [21]. The inconsistency in the occurrence of MDD could be due to differences in work-related characteristics, sleep quality, and burnout of the study populations [22]. Other possible explanations may be the diverse depression assessment tools and characteristics of the samples, e.g., ethnicity, gender, age group, and level of family or social support. The majority of our participants perceived that they had good social support from family, the head of the department, and psychiatrist peers. These supports may help alleviate distress, and lead to low depression scores in our study. In addition, most psychiatrists and psychiatry trainees reported an average level of loneliness, this could help affirm that they may receive adequate social support in life [21]. According to this, it could help explain why the prevalence of MDD was relatively low in our sample.

In this study, perception towards works was investigated using VAS (from 1 to 10), resulting in their perception levels sounding acceptable, as their median scores were from six to seven out of ten. Of note, the psychiatry trainees reported significantly lower perception levels of ability to control work schedule and income satisfaction as well as higher perception of work stress, compared to the psychiatrists. Additionally, perception of work stress and work satisfaction were found moderate correlation with MDD, particularly in the psychiatrist’s group. Our findings suggest that professional mental health care requires a sense of belonging and empowerment or workplace efficiency, a workplace with a sufficient budget and health care teams on duty, and a healthy work-life balance and good relationships among people are crucial in the workplace [23]. These needs should be the focus of healthcare policymakers and stakeholders [23].

From multiple regression analyses, similar to previous studies, loneliness was significantly associated with MDD in all samples [21]. Perception of work satisfaction and work stress were associated with contributing to MDD in psychiatrists [8, 13], while in psychiatry trainees perception of ability to control work schedule was significant. It is undeniable that working as a psychiatrist has a unique performance, because he/she has to establish a good doctor-patient relationship professionally – being a good communicator and listener [24], but relatively low income [25]. Furthermore, they may get extreme mental pressure and be at risk in clinical practice despite that there is a shortage of psychiatrists these days [13, 14, 26]. COVID-19 during and after pandemic also causes stressors to psychiatrists [17]. As a result, psychiatrists can feel an emotional burden [27]. Psychiatry trainees could also suffer from distress in the same way, although they have lower responsibility and shorter contact with people with illness, compared to psychiatrists. Although more trainees to become psychiatrists have been rapidly recruited, because of a small number of psychiatrists [26], the quality of training in psychiatrists remains crucial and should be standardized. However, they still have loads of work with closer supervision. This could therefore create psychological distress among psychiatry trainees as well.

In addition, having previous psychiatric illnesses, which were mostly MDD, was also a significant factor associated with depression in our samples. It could be that although they had been diagnosed with MDD, the treatment they received may have not been adequate to cure their depressive symptoms [28], they may still be suffering from the residual symptoms of MDD [29, 30], or they may refuse to seek help due to stigma [31]. Therefore, psychiatry trainees and psychiatrists with a history of psychiatric illnesses should receive close attention from their families, supervisors, and professional colleagues [32] to ensure that they receive adequate medical intervention in terms of both sufficient medication and robust psychosocial support. We also found that there were high frequencies of reported depressive symptoms including feeling tired, feeling down, lack of pleasure, having trouble concentrating, and having sleep problems. This information could help suggest monitoring the early warning signs of MDD among them. Notably, action is still required to reduce MDD rates in both psychiatrists and psychiatry trainees, although the prevalence of MDD in our study (Thailand) was not extremely high.

To our knowledge, this is the first study examining MDD and its associations such as work-related data, perception of social support, perceptions towards work, and loneliness. There were limitations in this study. Firstly, since the survey was conducted via an online platform with a response rate of 36.2%, we failed to get participants who are not interested in or not regularly involved in social media, such as people with MDD or with emotional distress. In addition, this study was an anonymous survey, making it impossible to identify the characteristics of responders versus non-responders. Therefore, it was unable to assess differences between the two groups to determine results in terms of generalization. As it was subject to selection bias (nonresponse bias), our sample might not represent the psychiatry trainees and psychiatrists at the national level. Secondly, although we advertised adequately (emphasizing that whether participating in the survey or not their decision will not affect their work/academic training), the number of psychiatry trainees is lower than expected, resulting in a lack of power in some data analyses. However, our research objectives were met. Thirdly, we did not collect current stressors which might help explain the MDD outcome. Lastly, this study might not include some possible confounding variables that can contribute to social support and loneliness. The study focused on social context factors rather than individual exposure factors, such as personal lifestyle, socio-economic status, and other mental health issues that could be aggravated by social or work-related conditions.

Therefore, future studies may seek to collect information about work, for example, different workplaces (e.g., rural or urban hospitals), types of welfare or insurance, and safety at the workplace. In addition, studying assertiveness to assist psychiatry trainees in getting a sense of control in workplaces, exploring factors linked to loneliness to alleviate emotional burden, and examining supporting factors to prevent distress, including focusing on individual exposure factors that may aggravated by social or work-related conditions may yield valuable knowledge that would inform effective targeted interventions for MDD in mental health care or other professionals. This study finding give us a clue to counter loneliness and work stress and improve job satisfaction for psychiatrist and psychiatric traineess for examples, developing systemic (policy level) or peer support group, those having depression requiring close supervision and improving training capacity to increase number of psychiatrists.

The findings of this research propose that the loneliness element, encompassing single, divorced, or widowed status, could intensify depression among psychiatrists and psychiatric trainees. Giving special attention to individuals in these categories may aid in averting adverse outcomes like suicide or self-harm. Furthermore, it is recommended to monitor individuals in this group for their perceptions of work, including aspects such as work schedule, work-related stress, and satisfaction with income.

## Conclusion

From the survey, 13.9% and 7.7% of psychiatrists and psychiatry trainees, respectively, had MDD. Among them, loneliness was a key contributing factor associated with MDD. Work satisfaction and work stress were significantly associated with MDD in psychiatrists, whereas in psychiatry trainees the perception of the ability to control work schedule was significant. Action against MDD among psychiatrists and psychiatry trainees needs to be taken into account, such as social support to prevent loneliness, increase work satisfaction, decrease work stress, or encourage flexibility in work schedule.

### Electronic supplementary material

Below is the link to the electronic supplementary material.

**Supplementary Material:** Supplementary Table 1, 2 and 3

## Data Availability

No datasets were generated or analysed during the current study.

## Abbreviations

MDD: Major depressive disorder
PHQ-9: Patient Health Questionnaire-9
QOL: Quality of life
RULS-6: The 6-item Revised UCLA Loneliness Scale
