# Supplementary material for: Depression among psychiatrists and psychiatry trainees and its associated factors regarding work, social support, and loneliness
Source: BMC Psychiatry. 2024 Feb 5;24:97. doi: 10.1186/s12888-024-05569-7 (PMC10840266; doi:10.1186/s12888-024-05569-7)
Supplement: Supplementary file 1 — Supplementary Material: Supplementary Table 1, 2 and 3 [file 12888_2024_5569_MOESM1_ESM.docx]

**Supplementary Table 1** Levels of depression, loneliness, and perception towards social supports and works (N=225)

| **Variables** | **Total**  (N=225); N (%) | **Psychiatrists**  (n=173); n (%) | **Psychiatry trainees**  (n=52); n (%) | **Chi^2^**  ***p*-value** |
| --- | --- | --- | --- | --- |
| **Depression** |  |  |  | 0.329 |
| Minimal | 143 (63.6) | 110 (63.6) | 33 (63.5) |  |
| Mild | 63 (28.0) | 46 (26.6) | 17 (32.7) |  |
| Moderate/severe | 19 (8.4) | 17 (9.8) | 2 (3.8) |  |
| **Loneliness** |  |  |  | 0.259 |
| Low | 0 |  |  |  |
| Average | 190 (84.4) | 143 (82.7) | 47 (90.4) |  |
| High | 35 (15.6) | 30 (17.3) | 5 (9.6) |  |
| **Perception towards social support** |  |  |  |  |
| **Family** |  |  |  | 0.526^a^ |
| Good | 199 (88.4) | 155 (89.6) | 44 (84.6) |  |
| Poor | 12 (5.3) | 8 (4.6) | 4 (7.7) |  |
| No answer | 14 (6.2) | 10 (5.8) | 4 (7.7) |  |
| **Chief of department** |  |  |  | 0.089 |
| Good | 149 (66.2) | 108 (62.4) | 41 (78.8) |  |
| Poor | 26 (11.6) | 22 (12.7) | 4 (7.7) |  |
| No answer | 50 (22.2) | 43 (24.9) | 7 (13.5) |  |
| **Psychiatrist friends** |  |  |  | 0.166 |
| Good | 178 (79.1) | 132 (76.3) | 46 (88.5) |  |
| Poor | 17 (7.6) | 15 (8.7) | 2 (3.8) |  |
| No answer | 30 (13.3) | 26 (15.0) | 4 (7.7) |  |
| **Perception towards works; Median (IQR)** |  |  |  |  |
| **Ability to control work schedule** | 7.0 (5, 8) | 7 (5, 8) | 6 (5, 7) | 0.023^b^ |
| Mean (SD) | 6.4 (1.9) |  |  |  |
| **Work stress** | 6.0 (5, 8) | 6 (4, 7) | 7 (5, 8) | 0.045^b^ |
| Mean (SD) | 5.9 (2.2) |  |  |  |
| **Income satisfaction** | 6.0 (5, 8) | 7 (5, 8) | 4 (2.8, 5.2) | <0.001^b^ |
| Mean (SD) | 6.0 (2.5) |  |  |  |
| **Work satisfaction** | 7.0 (6, 8) | 7 (6, 8) | 7 (6, 8) | 0.886^b^ |
| Mean (SD) | 7.0 (1.8) |  |  |  |

IQR: interquartile range; SD: standard deviation

Note:

^a^Fisher's exact test, ^b^Mann-Whitney U test

Level of depression using Patient Health Questionnaire-9 scores; Perception towards social support using self-rating whether ‘poor’ or ‘good’ of each item; Perception towards works using self-rating from 1-10 (extremely low – extremely high) of each item.

Each quartile of the lonelness score: Q1 (25th percentile) = 6, Q2 (50th percentile) = 9, Q3 (75th percentile) = 12

**Supplementary Table 2** Demographic, work-related characteristics, level of loneliness and perceptions towards social supports and works categorized by PHQ-9 (N=225)

| **Variables** | **Total** (N=225); N (%) | **PHQ-9**; n (%) | | ***p-value*** |
| --- | --- | --- | --- | --- |
|  |  | **Score <9**  (n=197) | **Score ≥9**  (n=28) |  |
| **Gender** |  |  |  | 0.159 |
| Male | 79 (35.1) | 73 (37.1) | 6 (21.4) |  |
| Female | 146 (64.9) | 124 (62.9) | 22 (78.6) |  |
| **Age (years)** |  |  |  | 0.768^a^ |
| Median (IQR) | 34 (30, 42) | 34 (30, 43) | 34 (30, 39.5) |  |
| **Marital status** |  |  |  | 1 |
| Single/Divorced | 146 (64.9) | 128 (65.0) | 18 (64.3) |  |
| Married | 79 (35.1) | 69 (35.0) | 10 (35.7) |  |
| **Number of children** |  |  |  | 0.77 |
| None | 176 (78.2) | 153 (77.7) | 23 (82.1) |  |
| One or more | 49 (21.8) | 44 (22.3) | 5 (17.9) |  |
| **Physical illness** |  |  |  | 0.629 |
| No | 134 (59.6) | 119 (60.4) | 15 (53.6) |  |
| Yes | 91 (40.4) | 78 (39.6) | 13 (46.4) |  |
| **Psychiatric illness** |  |  |  | 0.019^b^ |
| No | 206 (91.6) | 184 (93.4) | 22 (78.6) |  |
| Yes | 19 (8.4) | 13 (6.6) | 6 (21.4) |  |
| **Experience as a psychiatrist (years)** |  |  |  | 0.712^a^ |
| Median (IQR) | 6 (3, 14) | 7 (3, 14.2) | 5 (4, 9.2) |  |
| **Workplace** |  |  |  | 0.293 |
| Medical school | 105 (46.7) | 91 (46.2) | 14 (50.0) |  |
| Psychiatric hospital | 24 (10.7) | 19 (9.6) | 5 (17.9) |  |
| Other | 96 (42.7) | 87 (44.2) | 9 (32.1) |  |
| **Position** |  |  |  | 0.708 |
| General psychiatrist | 178 (79.5) | 157 (80.1) | 21 (75.0) |  |
| Child and adolescent psychiatrist | 46 (20.5) | 39 (19.9) | 7 (25.0) |  |
| **Work type** |  |  |  | 1^b^ |
| Only outpatient | 22 (9.8) | 20 (10.2) | 2 (7.1) |  |
| Outpatient and inpatient | 203 (90.2) | 177 (89.8) | 26 (92.9) |  |
| **Number of patients per day** |  |  |  | 0.249^a^ |
| Median (IQR) | 20 (10, 30) | 20 (10, 30) | 20 (15, 40) |  |
| **Working hours per week** |  |  |  | 0.740 |
| <40 | 57 (25.4) | 50 (25.5) | 7 (25.0) |  |
| 40-50 | 108 (48.2) | 96 (49.0) | 12 (42.9) |  |
| >50 | 59 (26.3) | 50 (25.5) | 9 (32.1) |  |
| **Number of night shifts per month** |  |  |  | 0.223^a^ |
| Median (IQR) | 6 (4, 10) | 6 (4, 10) | 5 (3.8, 8) |  |
| **Days off per month** |  |  |  | 0.937 |
| 0-2 | 21 (9.3) | 18 (9.1) | 3 (10.7) |  |
| 3-5 | 48 (21.3) | 41 (20.8) | 7 (25.0) |  |
| 6-8 | 101 (44.9) | 89 (45.2) | 12 (42.9) |  |
| >8 | 55 (24.4) | 49 (24.9) | 6 (21.4) |  |
| **Work position** |  |  |  | 0.345 |
| Psychiatrist | 173 (76.9) | 149 (75.6) | 24 (85.7) |  |
| Psychiatry trainee | 52 (23.1) | 48 (24.4) | 4 (14.3) |  |
| **Level of perceptions towards works** |  |  |  |  |
| **Income satisfaction** |  |  |  |  |
| Mean (SD) | 6.0 (2.5) |  |  |  |
| Median (IQR) | 6.0 (5, 8) | 7 (5, 8) | 5 (3, 6.2) | 0.004^a^ |
| **Work satisfaction** |  |  |  |  |
| Mean (SD) | 7.0 (1.8) |  |  |  |
| Median (IQR) | 7.0 (6, 8) | 7 (6, 8) | 5.5 (5, 6.2) | < 0.001^a^ |
| **Work stress** |  |  |  |  |
| Mean (SD) | 5.9 (2.2) |  |  |  |
| Median (IQR) | 6.0 (5, 8) | 6 (4, 7) | 7.5 (6.8, 8.2) | < 0.001^a^ |
| **Ability to control work schedule** |  |  |  |  |
| Mean (SD) | 6.4 (1.9) |  |  |  |
| Median (IQR) | 7.0 (5, 8) | 7 (5, 8) | 5 (3, 7) | < 0.001^a^ |
| **Perceived level of social support** |  |  |  |  |
| **Family** |  |  |  | 0.072^b^ |
| Good | 199 (88.4) | 176 (89.3) | 23 (82.1) |  |
| Poor | 12 (5.3) | 8 (4.1) | 4 (14.3) |  |
| No answer | 14 (6.2) | 13 (6.6) | 1 (3.6) |  |
| **Chief of department** |  |  |  | 0.448 |
| Good | 149 (66.2) | 133 (67.5) | 16 (57.1) |  |
| Poor | 26 (11.6) | 21 (10.7) | 5 (17.9) |  |
| No answer | 50 (22.2) | 43 (21.8) | 7 (25.0) |  |
| **Psychiatrist friends** |  |  |  | 0.933^b^ |
| Good | 178 (79.1) | 156 (79.2) | 22 (78.6) |  |
| Poor | 17 (7.6) | 15 (7.6) | 2 (7.1) |  |
| No answer | 30 (13.3) | 26 (13.2) | 4 (14.3) |  |
| **Loneliness** |  |  |  | 0.162^b^ |
| Average | 190 (84.4) | 169 (85.8) | 21 (75.0) |  |
| High | 35 (15.6) | 28 (14.2) | 7 (25.0) |  |

IQR: interquartile range; SD: standard deviation

Note:

^a^Mann-Whitney U test, ^b^Fisher's exact test

Patient Health Questionnaire-9 (PHQ-9) score ≥ 9 are considered as major depressive disorder

**Supplementary Table 3** Association of level of depression with demographic, work-related characteristics, level of loneliness and perceptions towards social supports and works categorized by PHQ-9 (N=225)

| **Demographic characteristics** | **Total** (N=225) | | **Psychiatrists** (n=173) | | **Psychiatry trainees** (n=52) | |
| --- | --- | --- | --- | --- | --- | --- |
|  | Median (IQR) | *p-value* | Median  (IQR) | *p-value* | Median (IQR) | *p-value* |
| **Gender** |  | 0.250^a^ |  | 0.450^a^ |  | 0.319^a^ |
| Male | 3 (1.0, 6.5) |  | 3 (2.0, 7.0) |  | 3 (1.0, 5.0) |  |
| Female | 3 (2.0, 6.0) |  | 3 (2.0, 6.0) |  | 4 (2.0, 7.0) |  |
| **Age (years)** | ρ = -0.10 | 0.148 | ρ = -1.74 | 0.022 | ρ = 0.22 | 0.122 |
| **Marital Status** |  | 0.368^a^ |  | 0.164^a^ |  | 0.254^a^ |
| Single/Divorce | 4 (2.0, 7.0) |  | 4 (2.0, 7.0) |  | 3 (2.0, 5.0) |  |
| Married | 3 (2.0, 6.0) |  | 3 (1.2, 5.8) |  | 7 (3.0, 7.0) |  |
| **Number of children** |  | 0.147^a^ |  | 0.137^a^ |  | NA |
| None | 3.5 (2.0, 7.0) |  | 3 (2.0, 7.0) |  | - |  |
| 1 or more | 3 (2.0, 4.0) |  | 3 (2.0, 4.2) |  | - |  |
| **Physical illness** |  | 0.533^a^ |  | 0.720^a^ | Mean (SD) | 0.525^c^ |
| No | 3 (2.0, 6.0) |  | 3 (2.0, 6.0) |  | 3.9 (2.9) |  |
| Yes | 3 (2.0, 7.0) |  | 3 (2.0, 7.0) |  | 4.4 (3.3) |  |
| **Psychiatric illness** |  | 0.127^a^ |  | 0.788^a^ |  | 0.009^a^ |
| No | 3 (2.0, 6.5) |  | 3 (2.0, 6.0) |  | 3 (2.0, 5.0) |  |
| Yes | 5 (2.5, 9.0) |  | 3 (2.0, 8.0) |  | 7 (6.0, 9.0) |  |
| **Workplace** |  | 0.655^b^ |  | 0.575^b^ |  | 0.608^a^ |
| Medical school | 3 (2.0, 7.0) |  | 3 (2.0, 7.0) |  | 4 (2.0, 5.0) |  |
| Psychiatric hospital | 3.5 (0.8, 8.0) |  | 4 (2.0, 8.0) |  | 3 (0, 7.0) |  |
| Others | 3 (2.0, 5.0) |  | 3 (2.0, 5.0) |  | - |  |
| **Position** |  | 0.385^a^ |  | 0.688^a^ |  | 0.311^a^ |
| General psychiatrist | 3 (2.0, 6.8) |  | 3 (2.0, 6.8) |  | 3 (1.8, 5.5) |  |
| Child and adolescent psychiatrist | 4 (2.0, 6.0) |  | 4 (2.0, 6.0) |  | 4 (3.0, 6.5) |  |
| **Work type** |  | 0.408^a^ |  | 0.401^a^ |  | NA |
| Only outpatient | 3 (2.0, 4.0) |  | 3 (2.0, 4.0) |  | - |  |
| Outpatient and inpatient | 3 (2.0, 7.0) |  | 3 (2.0, 7.0) |  | - |  |
| **Experience as a psychiatrist (years)** | ρ = -0.13 | 0.055 | ρ = -0.20 | 0.008 | ρ = 0.07 | 0.609 |
| **Number of patients per day** | ρ = 0.06 | 0.404 | ρ = 0.06 | 0.441 | ρ = 0.14 | 0.313 |
| **Working hours per week** |  | 0.429^b^ |  | 0.288^b^ | Mean (SD) | 0.542^d^ |
| <40 | 3 (1.0, 7.0) |  | 3 (1.0, 5.5) |  | 5.3 (4.3) |  |
| 40-50 | 3 (2.0, 6.0) |  | 3 (2.0, 6.0) |  | 3.8 (2.9) |  |
| >50 | 4 (2.0, 7.5) |  | 4 (2.0, 8.0) |  | 4.1 (2.7) |  |
| **Number of shifts per month** | ρ = -0.01 | 0.900 | ρ = 0.06 | 0.469 | ρ = -0.31 | 0.024 |
| **Days off per month** |  | 0.528^b^ |  | 0.283^b^ |  | 0.039 ^d^ |
| 0-2 | 2 (2.0, 5.0) |  | 4 (2.0, 8.0) |  | 1.6 (1.3) |  |
| 3-5 | 4 (2.0, 7.2) |  | 4 (2.0, 7.0) |  | 4.4 (3.1) |  |
| 6 or more | 3 (2.0, 6.0) |  | 3 (2.0, 5.0) |  | 4.5 (3.0) |  |
| **Death of patients who committed suicide** |  | 0.035^b^ |  | 0.024^b^ |  | NA |
| None | 4 (2.0, 7.0) |  | 4 (2.0, 7.0) |  | - |  |
| ≤ 1 month | 6 (4.0, 10.2) |  | 6 (4.0, 10.2) |  | - |  |
| > 1 month < 1 year | 4 (3.0, 7.0) |  | 4.5 (3.0, 7.0) |  | - |  |
| ≥ 1 year | 3 (1.0, 5.0) |  | 3 (1.0, 5.0) |  | - |  |

ρ = Spearman correlation coefficient; IQR: Interquartile range; PHQ-9: Patient Health Questionnaire-9

NA: not applicable (due to that number of psychiatry trainees in categorized variables (e.g., number of children, work type and death of patients who committed suicide) were not sufficient power for data analyses

Note:

^a^Mann-Whitney U test, ^b^Kruskal-Wallis test,  ^c^ t-test, ^d^ANOVA
